# Incidence of Out-of-Hospital Cardiac Arrest on a Postholiday Weekday

**DOI:** 10.1001/jamanetworkopen.2026.0832

**Published:** 2026-03-06

**Authors:** Min-Su Cha, Myoung-Je Song, Jong-Sun Kim

**Affiliations:** 1 Department of Emergency Medicine, International St Mary’s Hospital, Catholic Kwandong University College of Medicine, Incheon Metropolitan City, Republic of Korea; 2 Institute of Health and Medical Convergence Research, International St Mary’s Hospital, Catholic Kwandong University College of Medicine, Incheon Metropolitan City, Republic of Korea

## Abstract

**Importance:**

Although increased cardiovascular risk during holidays has been documented, whether this elevated risk persists into the postholiday transition period remains unclear.

**Objective:**

To quantify the incidence of out-of-hospital cardiac arrest on a postholiday weekday in South Korea.

**Design, Setting, and Participants:**

This nationwide cohort study analyzed data from the Out-of-Hospital Cardiac Arrest Surveillance database between January 1, 2013, and December 31, 2023. Adult out-of-hospital cardiac arrest cases occurring on weekdays were included, excluding those on holidays.

**Exposure:**

A postholiday weekday, defined as the first working day following any weekend or holiday period, compared with baseline weekdays (all other weekdays).

**Main Outcomes and Measures:**

The primary outcome was the out-of-hospital cardiac arrest incidence rate ratio (IRR) comparing a postholiday weekday with baseline weekdays, estimated using negative binomial regression. Subgroup analyses examined demographic and clinical factors, and additional analyses assessed dose-response associations according to the duration and type of preceding holidays.

**Results:**

Among the 203 471 adult participants (median [IQR] age, 71 [56-81] years; 130 348 [64.1%] male), 49 199 cases occurred on a postholiday weekday and 154 272 occurred on baseline weekdays. The daily out-of-hospital cardiac arrest incidence was significantly higher on a postholiday weekday than on baseline weekdays (median [IQR], 88 [78-98] vs 80 [71-89] cases), representing a 9% higher incidence (IRR, 1.09; 95% CI, 1.08-1.11; *P* < .001). Subgroup analysis revealed increased vulnerability among adults older than 65 years (IRR, 1.03; 95% CI, 1.02-1.04; *P* < .001), individuals with cardiac cause (IRR, 1.02; 95% CI, 1.01-1.04; *P* < .001), and those presenting with nonshockable rhythms (IRR, 1.03; 95% CI, 1.01-1.04; *P* < .001). A clear dose-response association was observed. Significant associations were observed after 2-day holidays (IRR, 1.10; 95% CI, 1.08-1.11; *P* < .001), 3-day holidays (IRR, 1.09; 95% CI, 1.03-1.15; *P* = .003), and holidays of 4 days or more (IRR, 1.10; 95% CI, 1.03-1.18; *P* = .007) but not after single-day holidays (IRR, 1.03; 95% CI, 0.98-1.08; *P* = .25). Weekend (IRR, 1.09; 95% CI, 1.07-1.11; *P* < .001) and mixed (IRR, 1.10; 95% CI, 1.06-1.14; *P* < .001) holidays were associated with a higher out-of-hospital cardiac arrest incidence, whereas public (IRR, 1.03; 95% CI, 0.97-1.09; *P* = .24) and temporary (IRR, 1.01; 95% CI, 0.89-1.13; *P* = .86) holidays were not.

**Conclusions and Relevance:**

In this nationwide cohort study, a postholiday weekday was associated with a significantly elevated out-of-hospital cardiac arrest incidence in South Korea, particularly after consecutive rest days and among vulnerable populations. These findings support enhanced emergency medical services preparedness, targeted public health messaging during holiday periods, and evaluation of preventive interventions for high-risk populations during postholiday transitions.

## Key Points

**Question:**

Is a postholiday weekday associated with a higher out-of-hospital cardiac arrest incidence vs baseline weekdays in South Korea?

**Findings:**

In this cohort study of 203 471 adult cases over 11 years, a postholiday weekday was associated with a 9% higher out-of-hospital cardiac arrest incidence than baseline weekdays. This phenomenon was particularly pronounced among older adults, individuals with cardiac cause, and after 2 or more consecutive holidays.

**Meaning:**

These findings suggest that the postholiday transition represents a period of increased cardiovascular vulnerability, underscoring the need for enhanced emergency medical preparedness during those times.

## Teaser

This cohort study examines the incidence of out-of-hospital cardiac arrest on a postholiday weekday vs baseline weekdays in South Korea.

## Introduction

Out-of-hospital cardiac arrest (OHCA) is a critical medical emergency and a major global public health challenge.^1^ It affects millions of individuals annually, with incidence rates ranging from 36.8 to 86.0 cases per 100 000 person-years across different regions.^2,3,4^ The annual OHCA incidence in South Korea has been reported to exceed 30 000 cases, with an age-standardized incidence rate of approximately 65.7 cases per 100 000 person-years as of 2023.^5^ Given the substantial burden of OHCA on public health systems and communities, understanding its temporal patterns is increasingly important for developing targeted prevention strategies and optimizing emergency response systems.

Holidays represent unique periods characterized by substantial deviations from routine daily life, potentially influencing the risk of cardiovascular events. During these periods, individuals are more likely to experience increased alcohol consumption, sleep disruption, and psychological stress.^6,7,8^ These patterns are reflected in population-level data, with multiple studies reporting elevated cardiovascular mortality during holidays.^9,10,11^ This phenomenon extends to South Korea, where OHCA incidence increases significantly during weekends and holidays compared with weekdays, persisting independently of cold temperatures and seasonal factors.^12,13^ Therefore, understanding the association could inform public health messaging, guide emergency medical service (EMS) resource allocation, and identify high-risk periods requiring enhanced preparedness.

A recent study^11^ from Singapore reported an increased OHCA incidence during holidays that persisted into the postholiday period, highlighting a previously underrecognized phenomenon. However, the postholiday phenomenon may vary substantially across geographic regions, ethnic populations, and cultural contexts and has not yet been examined in South Korea. Therefore, this study aimed to evaluate whether a postholiday weekday is associated with higher OHCA incidence using nationwide surveillance data spanning over a decade in South Korea.

## Methods

### Ethical Considerations

The institutional review board of Catholic Kwandong University approved this cohort study’s protocol and waived the requirement for individual consent, given the use of anonymized and publicly accessible data. This study was conducted and reported following the Strengthening the Reporting of Observational Studies in Epidemiology (STROBE) reporting guideline for cohort studies.^14^

### Study Setting

South Korea, with a population of approximately 51 million, operates a nationwide fire-based public EMS system consisting of 18 provincial fire departments and centralized dispatch centers under the National Fire Agency.^15^ Medical directors at dispatch centers provide online medical directions around the clock in response to EMS providers’ requests for services, including advanced airway management, fluid administration, cardiopulmonary resuscitation (CPR) withdrawal, drug administration, and complex problems. Patients with OHCA are typically transported to the nearest emergency department (ED), whereas those achieving prehospital return of spontaneous circulation are recommended to be transported to percutaneous coronary intervention-capable hospitals.

The South Korean calendar system includes standard weekends (Saturdays and Sundays) and several categories of holidays, including traditional, public, substitute, and temporary holidays. In this study, *holiday* was comprehensively defined as all nonworking days, encompassing both weekends and all holiday categories. The weekdays were classified into 2 exposure categories: (1) a postholiday weekday, defined as the single first working day immediately following any holiday period (to avoid confusion, this refers to the single 24-hour period starting at midnight of the first day after a holiday concludes), and (2) baseline weekdays, comprising all other working days. eTable 1 in Supplement 1 provides a detailed classification and enumeration of the holiday categories.

### Data Source

This study used the Out-of-Hospital Cardiac Arrest Surveillance (OHCAS) database—a legally mandated registry operated by the Korea Disease Control and Prevention Agency since 2006.^16^ The OHCAS is a nationwide registry that serves as a comprehensive source of standardized data on patients with OHCA in South Korea. This registry integrates the following 2 data sources: prehospital records from the National Fire Agency’s EMS and hospital-based clinical data from more than 700 participating medical institutions across all administrative districts. The initial dataset included basic demographics of patients with OHCA, dispatch times, witnessed status, bystander interventions, initial cardiac rhythm, and field resuscitation efforts. Moreover, the secondary data component involved medical record abstraction by trained experts using standardized data collection instruments, specifically those in the Utstein style guidelines and Resuscitation Outcomes Consortium Project framework.^17,18^ Hospital-based variables included ED visit time, resuscitation parameters, postresuscitation care, and clinical outcomes. Quality assurance measures comprised automated data validation checks, periodic audits by Korea Disease Control and Prevention Agency epidemiologists, and cross-verification between EMS and hospital records to ensure data completeness and accuracy. The registry’s mandatory reporting requirements and quality control processes, including automated validation and cross-verification, provide a robust foundation for epidemiological analysis.

### Study Participants

**Figure 1.**
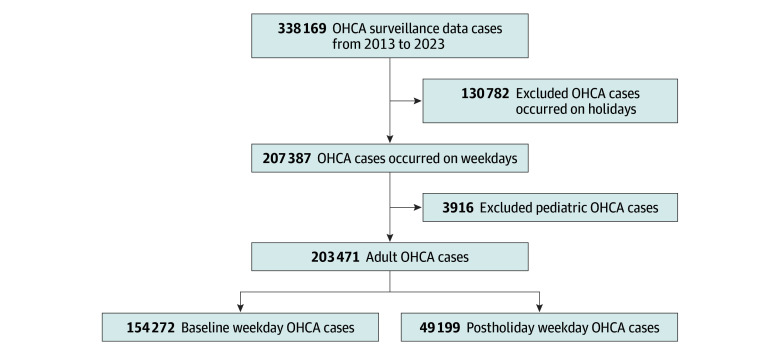
Flow Diagram of Study Population Selection OHCA indicates out-of-hospital cardiac arrest.

Overall, 338 169 OHCA cases were recorded in the OHCAS database between 2013 and 2023. We excluded 130 782 cases that occurred during holidays and 3916 pediatric patients younger than 18 years, given the distinct pathophysiological mechanisms and resuscitation protocols in this population (Figure 1).

### Variables and Measurements

Demographic variables included age, sex, and residential area (metropolitan vs nonmetropolitan or rural). Arrest characteristics included location (public vs nonpublic), witness status (witnessed vs unwitnessed), bystander CPR (performed vs not performed), and initial cardiac rhythm (shockable vs nonshockable).

The OHCAS database’s original classification of arrest cause distinguishes medical causes (including cardiac, respiratory, nontraumatic hemorrhage, terminal illness, sudden infant death syndrome, and other medical causes) from nonmedical causes (traumatic injuries and environmental exposures). We consolidated these into the following 6 groups to ensure statistical stability and minimize bias from sparse data: cardiac origin, traumatic injuries (traffic collisions, falls, blunt or penetrating trauma, and burns), asphyxiation (drowning, hanging, choking, and other forms of mechanical or environmental asphyxia), poisoning, other medical causes, and other nonmedical causes. Taxonomic restructuring ensured adequate event counts for robust estimation in the negative binomial regression models while grouping conditions with shared pathophysiological mechanisms.

### Study Outcomes

The primary objective was to quantify the differences in OHCA incidence between a postholiday weekday and baseline weekdays within South Korea’s calendar structure. This temporal pattern analysis was designed to generate epidemiological evidence to inform EMS resource allocation, guide targeted prevention strategies, and optimize public health interventions during high-risk transitional periods.

### Statistical Analysis

Normality was assessed using the Kolmogorov-Smirnov test and Q-Q plots. Continuous variables were summarized as medians (IQRs) and were compared using the Mann-Whitney *U* test due to their nonnormal distribution. Categorical variables were expressed as numbers (percentages) and compared using the χ^2^ test.

The incidence rate ratios (IRRs) with 95% CIs were estimated to quantify the association between the postholiday weekday and OHCA incidence. Incidence rates were calculated using the number of OHCA cases as the numerator and the annual midyear adult population of South Korea—obtained from the Korean Statistical Information Service—as the denominator (population at risk). Negative binomial regression was used instead of Poisson regression because of overdispersion (dispersion parameter >1.5), with log-transformed population as an offset term to account for demographic changes.

Subgroup analyses were performed by fitting separate negative binomial regression models for each stratum of the predetermined subgroups as follows: sex, age group, residential area, cause of arrest, initial cardiac rhythm, witness status, and bystander CPR. For each model, the IRR comparing the incidence of OHCA occurring on a postholiday weekday against baseline weekdays was estimated, with the log-transformed subgroup-specific population serving as an offset term. Patients with missing data were excluded from the subgroup analyses.

To assess dose-response associations, a postholiday weekday was categorized by the number of consecutive preceding nonworking days (1, 2, 3, or ≥4 days), with baseline weekdays (no preceding holiday) serving as the reference. OHCA incidence was also compared by preceding holiday type.

Statistical significance was set at 2-sided *P* < .05 for all analyses. Data management and statistical analyses were performed using R statistical software version 4.5.0 (R Project for Statistical Computing).

## Results

### Participants’ Baseline Characteristics and Clinical Information

Among the 203 471 adult participants analyzed (median [IQR] age, 71 [56-81] years; 130 348 [64.1%] male), 154 272 events occurred on baseline weekdays, and 49 199 events occurred on a postholiday weekday, with most events occurring in patients older than 65 years (91 079 events [59.0%] on baseline weekdays vs 29 009 events [58.9%] on a postholiday weekday) (Table 1). The clinical circumstances of arrest, including witness status, provision of bystander CPR, and initial cardiac rhythm, were comparable between the groups. Cardiac cause was the predominant cause and was slightly more frequent on a postholiday weekday than on baseline weekdays (35 240 patients [71.6%] vs 109 045 patients [70.6%]). A marginally higher proportion of cases occurred in metropolitan areas on a postholiday weekday than on baseline weekdays (17 773 patients [36.1%] vs 54 764 patients [35.4%]).

**Table 1.** Demographic and Clinical Characteristics of Participants

Characteristic	Patients, No. (%)		*P* value
	Baseline weekday (n = 154 272)	Postholiday weekday (n = 49 199)	
Age, median (IQR), y	71 (56-81)	70 (56-80)	.32
Age group, y			
18-35	8280 (5.3)	2520 (5.1)	.11
36-50	18 283 (11.8)	5827 (11.8)	
51-65	36 630 (23.7)	11 843 (24.0)	
>65	91 079 (59.0)	29 009 (58.9)	
Sex			
Female	55 324 (35.8)	17 799 (36.1)	.21
Male	98 948 (64.1)	31 400 (63.8)	
Residential area			
Metropolitan	54 764 (35.4)	17 773 (36.1)	.01
Nonmetropolitan or rural	99 508 (64.5)	31 426 (63.8)	
Witness status			
Witnessed	73 400 (47.5)	23 236 (47.2)	.36
Not witnessed	66 947 (43.3)	21 523 (43.7)	
Unknown or not recorded	13 925 (9.0)	4440 (9.0)	
Bystander cardiopulmonary resuscitation			
Performed	41 672 (27.0)	13 469 (27.3)	.26
Not performed	14 176 (9.1)	4466 (9.0)	
Unknown or not recorded	98 424 (63.7)	31 264 (63.5)	
Initial cardiac rhythm			
Shockable	14 168 (9.2)	4594 (9.4)	.61
Nonshockable	91 521 (59.8)	29 140 (59.6)	
Unknown or not recorded	47 341 (30.9)	15 104 (30.9)	
Arrest location			
Public	29 738 (19.2)	9161 (18.6)	<.001
Nonpublic	103 021 (66.7)	32 846 (66.7)	
Unknown or not recorded	21 513 (13.9)	7192 (14.6)	
Arrest cause			
Cardiac origin	109 045 (70.6)	35 240 (71.6)	<.001
Traumatic injury	20 264 (13.1)	6119 (12.4)	
Asphyxiation	5564 (3.6)	1618 (3.2)	
Poisoning	2161 (1.4)	686 (1.3)	
Other medical causes	7146 (4.6)	2332 (4.7)	
Other nonmedical causes	7804 (5.0)	2500 (5.0)	
Unknown or not recorded	2288 (1.4)	704 (1.4)	
Days of the week			
Monday	0	42 949 (87.2)	<.001
Tuesday	38 189 (24.7)	2140 (4.3)	
Wednesday	38 173 (24.7)	1440 (2.9)	
Thursday	38 560 (24.9)	1935 (3.9)	
Friday	39 350 (25.5)	735 (1.4)	

### Comparison of OHCA Incidence

**Figure 2.**
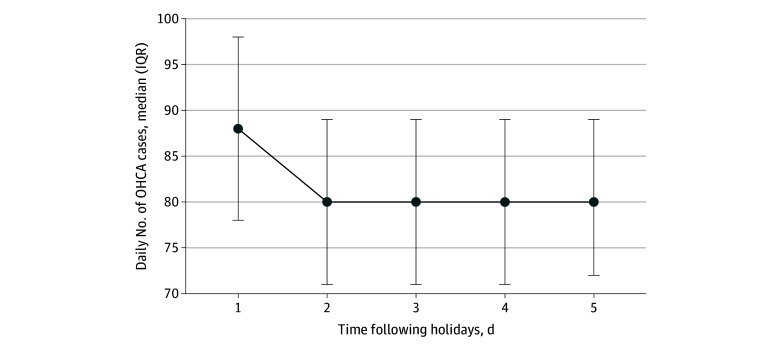
Graph of Temporal Decay of Out-of-Hospital Cardiac Arrest Incidence by Days Following Holidays Error bars denote IQRs. OHCA indicates out-of-hospital cardiac arrest.

The daily OHCA incidence during the study period (2013-2023) was significantly higher on a postholiday weekday than on baseline weekdays (median [IQR], 88 [78-98] cases vs 80 [71-89] cases). A postholiday weekday was associated with a 9% higher OHCA incidence than baseline weekdays (IRR, 1.09; 95% CI, 1.08-1.11; *P* < .001). This surge was confined to the first postholiday day (Figure 2).

### Subgroup Analysis

**Figure 3.**
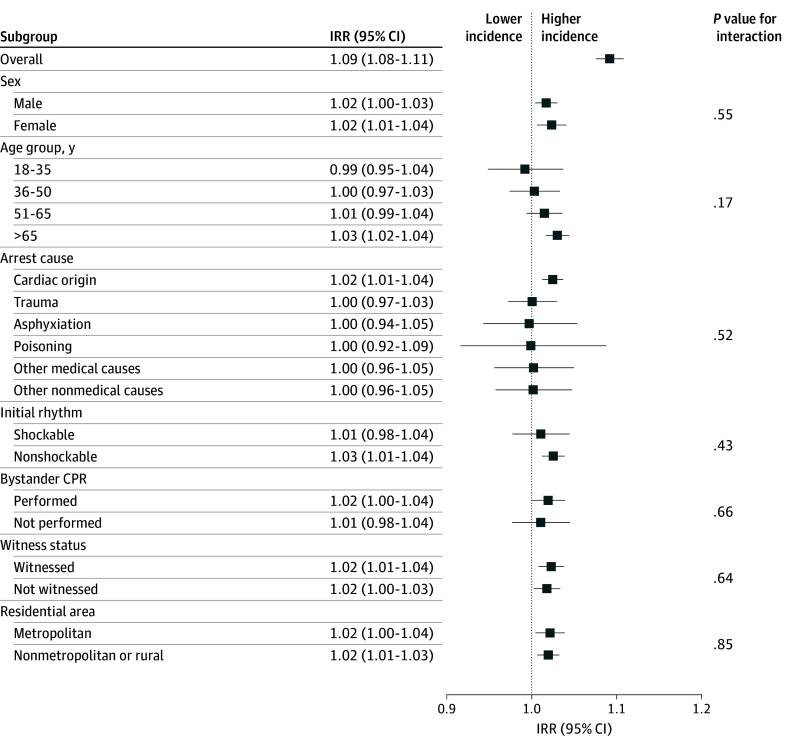
Forest Plot of Subgroup Analysis of Postholiday Weekday Association With Out-of-Hospital Cardiac Arrest Incidence CPR indicates cardiopulmonary resuscitation; IRR, incidence rate ratio.

The higher IRRs for OHCA incidence were consistent across sex, witness status, and residential area (Figure 3). However, heterogeneity was observed in other subgroups. The association was pronounced among adults older than 65 years (IRR, 1.03; 95% CI, 1.02-1.04; *P* < .001), those with cardiac cause (IRR, 1.02; 95% CI, 1.01-1.04; *P* < .001), and those presenting with nonshockable rhythms (IRR, 1.03; 95% CI, 1.01-1.04; *P* < .001).

Subgroup analyses comparing older (>65 years) and younger (≤65 years) adults demonstrated a higher OHCA incidence among witnessed arrests in the older adult group (IRR, 1.08; 95% CI, 1.05-1.12) (eFigure in Supplement 1). The older adult group showed a slightly higher witnessed rate than the younger adult group (58 742 patients [48.9%] vs 37 894 patients [45.4%]) (eTable 2 in Supplement 1), and clinical profiles remained stable throughout the 5-day postholiday period (eTable 3 in Supplement 1). These findings suggest that the surge in postholiday OHCA incidence among older adults represents a genuine epidemiological phenomenon, rather than ascertainment bias resulting from delayed discovery of unwitnessed arrests among solitary-dwelling older individuals.

### OHCA Risk by Duration and Type of Preceding Holidays

Regarding the duration of preceding holidays, the IRR for OHCA showed no significant association after single-day holiday compared with baseline weekdays (IRR, 1.03; 95% CI, 0.98-1.08; *P* = .25). However, significant associations were observed for all holiday durations lasting 2 days (IRR, 1.10; 95% CI, 1.08-1.11; *P* < .001), 3 days (IRR, 1.09; 95% CI, 1.03-1.15; *P* = .003), and 4 or more days (IRR, 1.10; 95% CI, 1.03-1.18; *P* = .007), suggesting a dose-response association (Table 2).

**Table 2.** Association of Postholiday Weekday With Out-of-Hospital Cardiac Arrest Incidence by Holiday Duration and Type

Variable	IRR (95% CI)	*P* value
Duration of preceding holidays, d		
1	1.03 (0.98-1.08)	.25
2	1.10 (1.08-1.11)	<.001
3	1.09 (1.03-1.15)	.003
≥4	1.10 (1.03-1.18)	.007
Type of preceding holidays		
Public holidays	1.03 (0.97-1.09)	.24
Temporary holidays	1.01 (0.89-1.13)	.86
Weekends	1.09 (1.07-1.11)	<.001
Mixed holidays^a^	1.10 (1.06-1.14)	<.001

Abbreviation: IRR, incidence rate ratio.

^a^ Mixed holidays are defined as consecutive periods that incorporate different types of holidays, including traditional holidays, substitute holidays, weekends, and public holidays.

When stratified by preceding holiday type, traditional and substitute holidays did not occur in isolation during the study period and were invariably contiguous with other holiday types. Therefore, they were classified under the mixed holiday category for analysis. A postholiday weekday following weekends (IRR, 1.09; 95% CI, 1.07-1.11; *P* < .001) and mixed holidays (IRR, 1.10; 95% CI, 1.06-1.14; *P* < .001) was associated with a higher OHCA incidence, whereas no significant associations were observed for a postholiday weekday following public (IRR, 1.03; 95% CI, 0.97-1.09; *P* = .24) or temporary (IRR, 1.01; 95% CI, 0.89-1.13; *P* = .86) holidays.

## Discussion

This cohort study examined the temporal patterns of OHCA incidence in association with postholiday periods using comprehensive nationwide surveillance data from South Korea. OHCA incidence was 9% higher on a postholiday weekday than on baseline weekdays, with a higher incidence observed after 2 or more consecutive rest days. This postholiday phenomenon was particularly pronounced among older adults and patients with cardiac cause, suggesting specific vulnerable populations that can benefit from targeted preventive interventions.

Previous studies^9,11,13^ have consistently reported higher cardiac mortality during holidays in Western populations, Singapore, and South Korea. Notably, our study provides novel evidence that this higher incidence is not limited to holidays but is also observed during the postholiday transition period. The magnitude of the association observed in our study (IRR, 1.09) was comparable to previously reported elevated incidence during weekends and holidays, highlighting the postholiday transition as an equally critical and previously underrecognized window of cardiovascular vulnerability.

The higher postholiday OHCA incidence observed is likely multifactorial, reflecting a complex interplay between biological triggers and behavioral shifts. Biologically, the abrupt transition from a period of rest to the resumption of structured daily routines may induce substantial physiological stress. This shift is usually associated with a surge in sympathetic nervous system activity and catecholamine release, which can promote platelet aggregation, increase myocardial oxygen demand, and trigger fatal arrhythmias or plaque rupture in vulnerable individuals.^8,13,19^ Behaviorally, holiday periods usually involve cumulative lifestyle disruptions. Alcohol consumption may be a particularly important factor, as it can precipitate arrhythmias. Excessive consumption of alcohol has been associated with increased risk of atrial fibrillation, atrial flutter, and sudden cardiac death, even among individuals without preexisting structural heart disease.^20,21^ The underlying mechanisms include direct electrophysiological alterations (shortened atrial refractory periods and QT prolongation), autonomic imbalance, and electrolyte disturbances, which collectively may contribute to the increased risk of sudden cardiac death.^20,21^ Sleep pattern disruption is also an important factor. A prospective cohort study^22^ demonstrated that individuals with the highest day-to-day variability in sleep duration or timing had more than 2-fold increased risk of cardiovascular events, potentially mediated through disrupted autonomic nervous system rhythms. Suboptimal medication adherence during festivities, combined with a tendency for patients to postpone seeking medical attention for prodromal symptoms until the holidays end (health care–seeking delay), may lead to a concentration of critical cardiovascular events on the first postholiday weekday. Ultimately, these biological and behavioral changes lead to an accumulated physiological debt, creating a vulnerable window that precipitates the observed surge in OHCA incidence.

Not all studies have reported consistent findings. Lennon et al^23^ analyzed extensive Australian data spanning from 1989 to 2015, encompassing over 700 000 cardiovascular and 250 000 stroke deaths, and found nonsignificant differences in holiday mortality of 1.08% (*P* = .35) and 0.20% (*P* = .87), respectively. Similarly, Lin et al^24^ examined 11 946 cases of traumatic injury mortality over a decade in Taipei and reported no association with in-hospital mortality during weekends (*P* = .66) or holiday seasons (*P* = .71). These discrepancies reflect fundamental methodological differences. Our study examined OHCA incidence in the postholiday period, whereas prior studies assessed in-hospital mortality during holidays, differing in both end point and target period.^23,24^ Therefore, the findings of those prior studies do not negate our observation of a higher OHCA incidence on a postholiday weekday.

Subgroup analysis revealed that a postholiday weekday was consistently associated with higher OHCA incidence across sex, witness status, and residential area. However, no significant associations were observed in either subgroup when stratified by bystander CPR. This null finding should be interpreted cautiously, as bystander CPR data showed substantial missingness, with most cases lacking this information in both the baseline weekday and postholiday weekday cohorts. The extensive exclusion of cases because of missing data likely limited the statistical power to detect meaningful associations within bystander CPR subgroups. Another plausible explanation is that bystander CPR is largely associated with situational and environmental factors—including location, witness availability, and public awareness—rather than the postholiday transition.

Differential associations between a postholiday weekday and OHCA incidence were observed according to age, cause, and initial cardiac rhythm. This finding aligns with established evidence that older adults with cardiovascular disease experience a high prevalence of geriatric conditions—including multimorbidity, polypharmacy, and frailty—all of which are associated with reduced physiological reserve and stress tolerance.^25,26^ Specifically, the older population may have a limited ability to cope with the physiological demands of transitioning between prolonged rest periods and regular activity resumption. The postholiday association observed exclusively in the nonshockable rhythm subgroup is consistent with prior findings of circaseptan variability limited to unwitnessed arrests with nonshockable rhythms.^10^ However, the mechanisms underlying this pattern remain unclear. Although ventricular fibrillation, categorized as a shockable rhythm, typically indicates acute coronary occlusion and theoretically aligns with holiday-related cardiac stress, it accounts for only approximately 23% of all cardiac arrests.^27,28,29^ The proportion of patients presenting with shockable rhythms in our study cohort was even lower. This limited sample size may have constrained the statistical power to detect a postholiday association in the shockable rhythm subgroup despite its presumed biological plausibility.

The duration and type of preceding holidays are important factors associated with OHCA incidence on a postholiday weekday. Significant associations were observed after other types of holidays, but not after public or temporary holidays. This differential pattern is likely related to holiday duration, as public and temporary holidays in South Korea typically occur as isolated single-day events rather than consecutive multiday periods. Furthermore, the threshold phenomenon observed on 2 consecutive days suggests that brief routine interruptions are insufficient to trigger cumulative lifestyle disruptions and subsequent physiological stress related to the transition back to work.

### Strengths and Limitations

A strength of this study is that the use of a comprehensive national OHCA registry spanning 11 years allows for exceptional statistical power and population representativeness, capturing more than 200 000 adult OHCA cases across diverse geographic and demographic contexts. Standardized data collection protocols facilitate high-quality and internationally comparable outcome measures. However, this study had some limitations. First, the observational design precludes causal inference. Second, for unwitnessed arrests, the exact time of death and duration of delay-to-discovery remain uncertain, which may limit the ability to definitively rule out an artifact resulting from delayed discovery. However, our subgroup analysis of adults older than 65 years suggested that this bias was likely minimal. Third, some variables had a high proportion of missing values, including bystander CPR and initial cardiac rhythm. Fourth, our study could not account for individual-level personal leave or vacations taken outside of the official South Korean holiday system, potentially biasing our results. However, given that national holidays involve a mass-synchronized transition of the entire society, their impact likely outweighs the influence of individual-level holiday patterns.

## Conclusions

A postholiday weekday was associated with a significantly higher OHCA incidence than baseline weekdays. This association was particularly pronounced among older adults, individuals with cardiac cause, and those presenting with nonshockable rhythms after 2 or more consecutive holidays. These findings have important implications for public health planning and EMS preparedness, suggesting the need for enhanced resource allocation and heightened clinical vigilance during the postholiday period. Future research should investigate factors associated with changes in postholiday OHCA incidence, including sleep pattern alterations, alcohol consumption, emotional stress, and medication adherence.

## Notes

Supplement 1. eTable 1. Calendar system and holidays in South Korea (2013–2023) eTable 2. Baseline characteristics and clinical profiles of patients with out-of-hospital cardiac arrest, stratified by age group (older [>65 years] vs. younger [≤65 years] adults) eTable 3. Out-of-hospital cardiac arrest characteristics among older adults (>65 years) across post-holiday days eFigure. Age-stratified (younger [≤65 years] and older [>65 years] adults) subgroup analysis of out-of-hospital cardiac arrest incidence on post-holiday weekday

Supplement 2. Data Sharing Statement
