# Errors in Figure 1, Abstract, and Results

**DOI:** 10.1001/jamanetworkopen.2026.10292

**Published:** 2026-04-08

In the Original Investigation titled “Incidence of Out-of-Hospital Cardiac Arrest on a Postholiday Weekday,”^1^ published March 6, 2026, there was an error in Figure 1. The number of postholiday weekday OHCA cases was incorrectly listed as 149 199. The correct number is 49 199. Also, the *P* value for holidays of 4 days or more was incorrect in both the abstract and results. It was incorrectly shown as *P* = .008 but the correct number is *P* = .007. This article has been corrected.^1^
