# Supplementary material for: Incidence of Out-of-Hospital Cardiac Arrest on a Postholiday Weekday
Source: JAMA Netw Open. 2026 Mar 6;9(3):e260832. doi: 10.1001/jamanetworkopen.2026.0832 (PMC12966924; doi:10.1001/jamanetworkopen.2026.0832)
Supplement: Supplement 1. — eTable 1. Calendar system and holidays in South Korea (2013–2023) eTable 2. Baseline characteristics and clinical profiles of patients with out-of-hospital cardiac arrest, stratified by age group (older [>65 years] vs. younger [≤65 years] adults) eTable 3. Out-of-hospital cardiac arrest characteristics among older adults (>65 years) across post-holiday days eFigure. Age-stratified (younger [≤65 years] and older [>65 years] adults) subgroup analysis of out-of-hospital cardiac arrest incidence on post-holiday weekday [file jamanetwopen-e260832-s001.pdf]

Supplemental Online Content

Cha MS, Song MJ, Kim JS. Incidence of out-of-hospital cardiac arrest on a postholiday weekday. *JAMA Netw Open*. 2026;9(3):e260832. doi:10.1001/jamanetworkopen.2026.0832

eTable 1. Calendar system and holidays in South Korea (2013–2023)

eTable 2. Baseline characteristics and clinical profiles of patients with out-of-hospital cardiac arrest, stratified by age group (older [>65 years] vs. younger [≤65 years] adults)

eTable 3. Out-of-hospital cardiac arrest characteristics among older adults (>65 years) across post-holiday days

eFigure. Age-stratified (younger [≤65 years] and older [>65 years] adults) subgroup analysis of out-of-hospital cardiac arrest incidence on post-holiday weekday

This supplemental material has been provided by the authors to give readers additional information about their work.

© 2026 Cha MS et al. *JAMA Network Open*.

eTable 1. Calendar system and holidays in South Korea (2013–2023)

Category	Description/Example dates	Number of days
Public holidays	New Year's Day (January 1)	11
	Independence Movement Day (March 1)	11
	Buddha's Birthday (Lunar April 8)	11
	Children's Day (May 5)	11
	Memorial Day (June 6)	11
	Liberation Day (August 15)	11
	National Foundation Day (October 3)	11
	Hangul Proclamation Day (October 9)	11
	Christmas Day (December 25)	11
Traditional holidays	Lunar New Year Holidays (3 days around January 1 of the Lunar calendar)	33
	Korean Thanksgiving Day (3 days around August 15 of the Lunar calendar)	33
Substitute holidays	Weekdays designated as holidays when national holidays and traditional festivals overlap with weekends	16
Temporary holidays	Ad hoc holidays designated by the government for special circumstances (e.g., presidential or local elections and commemorative events)	9
Weekends	Every Saturday and Sunday	1148

© 2026 Cha MS et al. *JAMA Network Open*.

eTable 2. Baseline characteristics and clinical profiles of patients with out-of-hospital cardiac arrest, stratified by age group (Older [>65 years] vs. Younger [≤65 years] adults)

	Older adults (n=120,088)	Younger adults (n=83,383)	*P*-value
Age (years)	79 (73–85)	53 (44–60)	<.001
Age groups			<.001
18–35	0 (0.0)	10,800 (12.9)	
36–50	0 (0.0)	24,110 (28.9)	
51–65	0 (0.0)	48,473 (58.1)	
>65	120,088 (100.0)	0 (0.0)	
Female	51,760 (43.1)	21,363 (25.6)	<.001
Residential area			<.001
Metropolitan	41,435 (34.5)	31,102 (37.3)	
Non-metropolitan/rural	78,653 (65.4)	52,281 (62.6)	
Witness status			<.001
Witnessed	58,742 (48.9)	37,894 (45.4)	
Not witnessed	50,897 (42.3)	37,573 (45.0)	
Unknown/not recorded	10,449 (8.7)	7,916 (9.4)	
Bystander CPR			<.001
Performed	31,484 (26.2)	23,657 (28.3)	
Not performed	10,562 (8.7)	8,080 (9.6)	
Unknown/not recorded	78,042 (64.9)	51,646 (61.9)	
Initial cardiac rhythm			<.001
Shockable	7,209 (6.0)	11,553 (13.9)	
Non-shockable	74,978 (63.0)	45,683 (55.0)	
Unknown/not recorded	36,748 (30.8)	25,697 (30.9)	
Arrest location			<.001
Public	15,318 (12.7)	23,581 (28.2)	
Non-public	87,116 (72.5)	48,751 (58.4)	
Unknown/not recorded	17,654 (14.7)	11,051 (13.2)	
Arrest cause			<.001

© 2026 Cha MS et al. *JAMA Network Open*.

Cardiac origin	97,347 (81.0)	46,938 (56.2)	
Traumatic injury	8,183 (6.8)	18,200 (21.8)	
Asphyxiation	4,265 (3.5)	2,917 (3.4)	
Poisoning	938 (0.7)	1,909 (2.2)	
Other medical causes	5,074 (4.2)	4,404 (5.2)	
Other non-medical causes	2,952 (2.4)	7,352 (8.8)	
Unknown/not recorded	1,329 (1.1)	1,663 (1.9)	
Days of the week			.30
Monday	25,273 (21.0)	17,676 (21.1)	
Tuesday	23,675 (19.7)	16,654 (19.9)	
Wednesday	23,444 (19.5)	16,169 (19.3)	
Thursday	24,049 (20.0)	16,446 (19.7)	
Friday	23,647 (19.6)	16,438 (19.7)	

Continuous variables are presented as median (interquartile range)
Categorical variables are presented as numbers (percentages).
Abbreviation: CPR, cardiopulmonary resuscitation

© 2026 Cha MS et al. *JAMA Network Open*.

eTable 3. Out-of-hospital cardiac arrest characteristics among older adults (>65 years) across post-holiday day

	Number of cases	OHCA incidence	Witnessed	Non-shockable
Day 1	29,009	51 (43–59)	14,111 (48.6%)	18,076 (62.3%)
Day 2	25,390	46 (40–53)	12,424 (48.9%)	15,874 (62.5%)
Day 3	23,486	46 (40–55)	11,524 (49.1%)	14,710 (62.6%)
Day 4	22,021	47 (39–55)	10,854 (49.3%)	13,759 (62.5%)
Day 5	20,182	46 (40–55)	9,829 (48.7%)	12,559 (62.2%)

Continuous variables are presented as median (interquartile range)
Categorical variables are presented as numbers (percentages).
Abbreviation: OHCA, out-of-hospital cardiac arrest

© 2026 Cha MS et al. *JAMA Network Open*.

eFigure. Age-stratified (younger [≤65 years] and older [>65 years] adults) subgroup analysis of out-of-hospital cardiac arrest incidence on post-holiday weekday

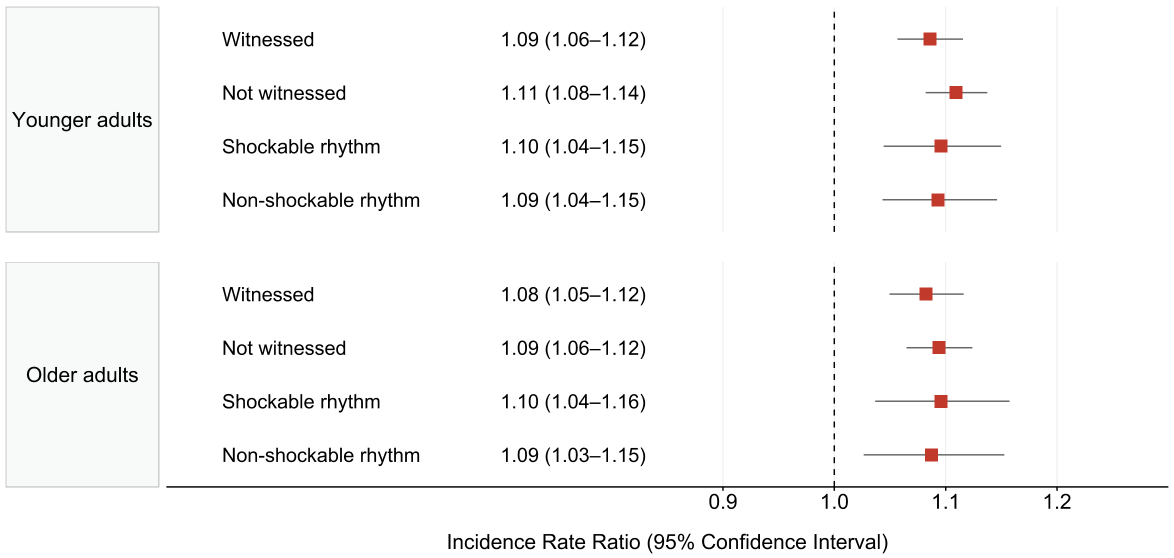

© 2026 Cha MS et al. *JAMA Network Open*.
