# Supplementary material for: Incidence of Out-of-Hospital Cardiac Arrest on a Postholiday Weekday
Source: JAMA Netw Open. 2026 Mar 6;9(3):e260832. doi: 10.1001/jamanetworkopen.2026.0832 (PMC12966924; doi:10.1001/jamanetworkopen.2026.0832)
Supplement: Supplement 2. — Data Sharing Statement [file jamanetwopen-e260832-s002.pdf]

Data Sharing Statement

Cha. Incidence of Out-of-Hospital Cardiac Arrest on a Postholiday Weekday. *JAMA Netw Open*. Published March 06, 2026. doi:10.1001/jamanetworkopen.2026.0832

Data
Data available: No

Additional Information
Explanation for why data not available: The data are publicly available.
